# Inter- and intraindividual variability in daily resting heart rate and its associations with age, sex, sleep, BMI, and time of year: Retrospective, longitudinal cohort study of 92,457 adults

**DOI:** 10.1371/journal.pone.0227709

**Published:** 2020-02-05

**Authors:** Giorgio Quer, Pishoy Gouda, Michael Galarnyk, Eric J. Topol, Steven R. Steinhubl

**Affiliations:** 1 Scripps Research Translational Institute, La Jolla, California, United States of America; 2 University of Alberta, Division of Cardiology, Edmonton, Alberta, Canada; Texas A&M University, UNITED STATES

## Abstract

**Background:**

Heart rate is routinely measured as part of the clinical examination but is rarely acted upon unless it is well outside a population-based normal range. With wearable sensor technologies, heart rate can now be continuously measured, making it possible to accurately identify an individual’s “normal” heart rate and potentially important variations in it over time. Our objective is to describe inter- and intra-individual variability in resting heart rate (RHR) collected over the course of two years using a wearable device, studying the variations of resting heart rate as a function of time of year, as well as individuals characteristics like age, sex, average sleep duration, and body mass index (BMI).

**Methods and findings:**

Our retrospective, longitudinal cohort study includes 92,457 de-identified individuals from the United States (all 50 states), who consistently—over at least 35 weeks in the period from March 2016 to February 2018, for at least 2 days per week, and at least 20 hours per day—wore a heart rate wrist-worn tracker. In this study, we report daily RHR and its association with age, BMI, sex, and sleep duration, and its variation over time. Individual daily RHR was available for a median of 320 days, providing nearly 33 million daily RHR values. We also explored the range in daily RHR variability between individuals, and the long- and short-term changes in the trajectory of an individual’s daily RHR.

Mean daily RHR was 65 beats per minute (bpm), with a range of 40 to 109 bpm among all individuals. The mean RHR differed significantly by age, sex, BMI, and average sleep duration. Time of year variations were also noted, with a minimum in July and maximum in January. For most subjects, RHR remained relatively stable over the short term, but 20% experienced at least 1 week in which their RHR fluctuated by 10 bpm or more.

**Conclusions:**

Individuals have a daily RHR that is normal for them but can differ from another individual’s normal by as much as 70 bpm. Within individuals, RHR was much more consistent over time, with a small but significant seasonal trend, and detectable discrete and infrequent episodes outside their norms.

## Introduction

Measuring an individual’s heart rate is a routine part of a clinical examination. Reference ranges for what is considered a “normal” heart rate have been established based on single determinations in artificial settings, such as the US National Health and Nutrition Examination Survey [1]. These population ranges are rarely a helpful measure of individual health, as heart rates that fall within the normal range have been associated with increased risks of mortality and morbidity [2–4]. While a high value of resting heart rate (RHR) is in general associated with increased cardiovascular risk in both general populations [5–7] and in those with cardiovascular comorbidities [8–11], one study has shown a low RHR (<65 bpm) to be associated with higher cardiovascular risk [12]. In this same study, a significant change in RHR at 3 years of follow-up was associated with a higher risk of cardiovascular disease. Thus, a single measurement of heart rate provides very little useful information about the current health of an individual, unless well out of the expected range of normal. Longitudinal, individual data may be of more value, as several studies of changes in discrete heart rate measurements over long periods of time have found an association with cardiovascular outcomes [13–16]. A long view of individual changes in cardiac performance may provide useful information to help refine existing phenotypes of health and illness and, when combined with other standard or enhanced clinical tests, might detect diseases at their earliest stages [17–19].

Today, through the integration of photoplethysmography (PPG) sensors into a range of commercial wearable sensors, heart rate can be measured continuously over the lifespan. Their accuracy at present is similar to that of standard electrocardiography (ECG) monitoring, especially at rest [20–22]. The ubiquity of these sensors enables a unique opportunity to better understand how RHR varies over time for and between individuals over the span of days, weeks, years, and eventually, lifetimes.

Only with such understanding can we begin to explore the myriad of environmental insults or physiologic changes that can impact heart rate. Unlike moment-to-moment changes in heart rate, an individual’s RHR, when measured daily in a consistent setting, might provide a measure of an individual’s overall cardiovascular physiologic status. Changes over weeks to months might indicate changes in cardiovascular fitness [23], but changes over days might reflect infection or other significant physiologic triggers.

In the present study, we retrospectively explore the largest dataset of longitudinal daily RHR available, including over 92,000 individuals who routinely used a wearable activity and heart-rate monitoring device over at least 35 weeks, for at least 2 days per week, and at least 20 hours per day. This dataset accounts for almost 33 million person-days of data for examining inter- and intraindividual variations in RHR. We analysed the association of the individual mean RHR, as well as RHR variability, with age, body-mass index (BMI), and average time asleep. We also explored the range in day-to-day RHR variability between individuals, and the long- and short-term changes in the trajectory of an individual’s daily RHR.

## Methods

### Study design

This was a retrospective, observational cohort study that used data collected from individuals wearing one of the following Fitbit devices: Charge HR versions 1–3 (65% of individuals), Blaze (21%), Alta HR (8%), Ionic (3%), Surge (2%), or Versa (1%). These devices continuously monitor heart rate every second as well as track and report the daily total duration of sleep. Activity data were not available for this analysis.

### Population

**Fig 1:**
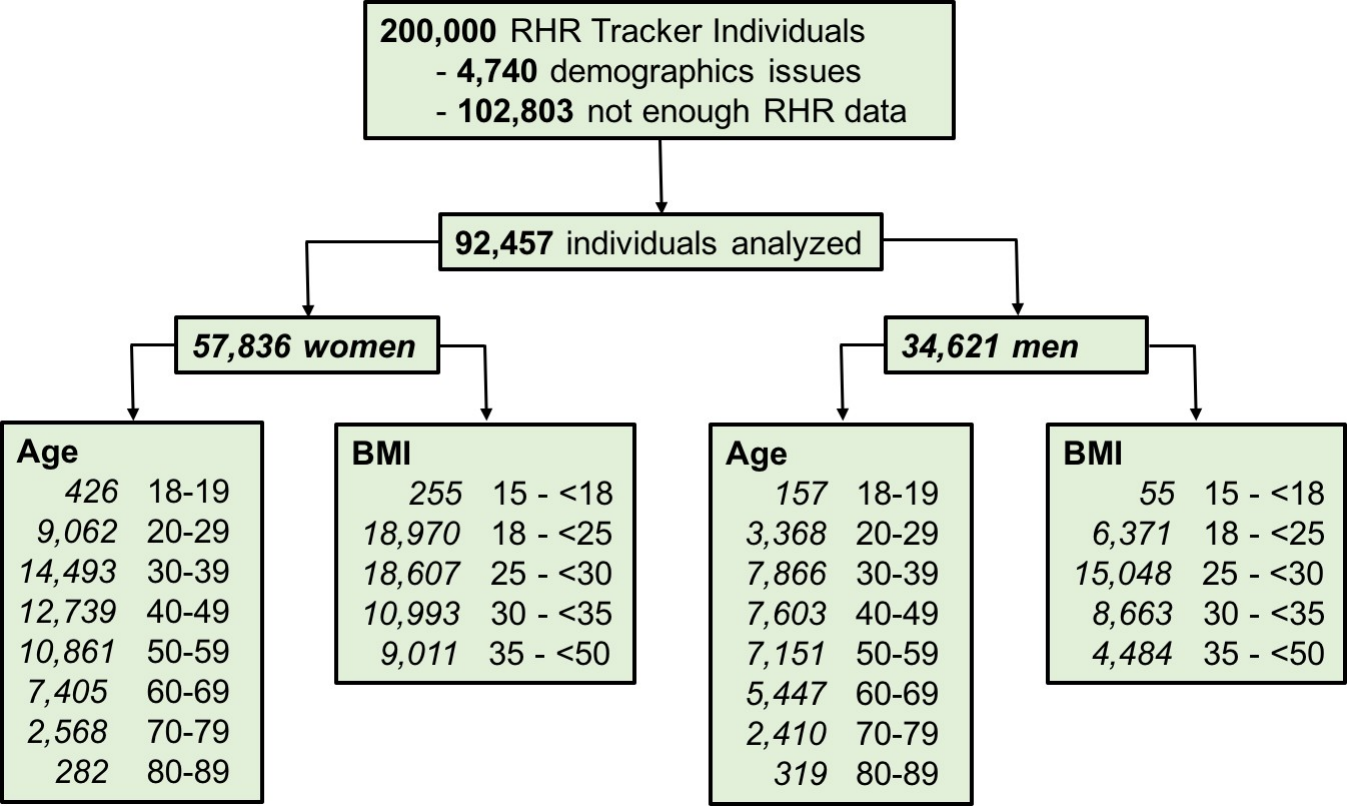
Subject selection for the study. We first removed users of resting heart rate (RHR) tracking devices based on demographic characteristics: age under 18 years, no sex specified, or body-mass index (BMI) outside the range of 15–50 kg/m^2^. Then we removed users who did not have at least 2 days per week of use, with the device worn 20 hours or more per day, for a minimum of 35 weeks. The demographic and clinical data for the resulting cohort divided by gender are shown.

The initial dataset included a convenience sample of 200,000 persons consistently using the device, from the United States—from all 50 states and the District of Columbia—and a total of 65,153,836 daily RHR measurements that had been collected between March 2016 and February 2018. We then excluded users who did not report their sex, whose self-reported age was under 18 years or over 100 years, and whose BMI was outside the range of 15–50 kg/m^2^ (calculated from self-reported height and weight), assuming the latter were more likely to be erroneous. Furthermore, we considered only measurements of the RHR for days in which the device was worn for at least 20 hours, with the wrist-worn time determined by the device based on the data collected. Doing so helped assure that RHR was calculated similarly for all individuals for all days. The final analysis population included 92,457 individuals with at least two RHR measurement days per week for a minimum of 35 weeks. The effects of the selection criteria, together with the demographic characteristics of the individuals in our datasets (in terms of sex, age, and BMI categories), are described in Fig 1.

### Ethical considerations

The protocol for this study was reviewed by the Scripps Institutional Review Board and deemed exempt from human-subject research protections. The data accessed for this study contained only aggregated, non-personally identifiable information. Fitbit users are made aware that their de-identified data could potentially be used for research in the Fitbit Privacy Policy.

### Variables and statistical analysis

RHR was measured using interbeat intervals derived from the PPG sensor. This measure has been demonstrated to have good agreement with electrocardiography-based RHR measured during sleep [24]. RHR was calculated by a proprietary formula from Fitbit, which provides for each individual a single value each day. This value is expected to be representative of the true value of RHR that would be obtained in a supine position immediately after waking but before getting out of bed. The value of the RHR reported by the devices used in this study are in agreement with the ones from a chest-based consumer electrocardiogram [25, 26]. In another study, wrist-based device heart rate determination has been shown to have a <5% error in a range of devices and activities relative to gold-standard, and closer to 1% when at rest [21]. To determine the significance of any difference in average RHR between groups based on age, BMI, and hours of sleep, we used a Welch’s *T*-test to compare means of two independent samples.

The fraction of variance in the interindividual variability of the mean RHR that depends on a specific variable was obtained by calculating the coefficient of determination (*R*^2^). In particular, since the relationship between the independent variables (age, BMI, and hours of sleep) and the dependent variable (mean RHR) is not linear (as shown in the results), we opted for treating each independent variable as a categorical variable. For each independent variable (among age, BMI, and sleep), we divided the population into n = 15 groups based on percentiles (e.g., the first group includes all individuals such that the independent variable is smaller than or equal to the 100/n percentile). Then we calculated the mean of the dependent variable (RHR) among all individuals in each group, and we kept this value as our best predictor of the mean RHR for individuals in that group. These values (one per group) were then used to calculate the sum of squares of residuals (the distance between the predicted RHR and the mean RHR for a single individual). Finally, the coefficient of determination is equal to one minus the total sum of squares of the distance between the predicted value of the RHR and the mean RHR over all individuals.

We determined the fluctuation of RHR over time of year by dividing the whole year into 52 weeks, with Week 1 corresponding to the week from January 1 to January 7 of each year in the dataset. For each individual, we calculated the average RHR corresponding to each week of the year by considering all data available for that week. If an individual had not collected any data for a given week, we used linear interpolation for circular data to calculate the expected RHR for that week. The median number of weeks interpolated for each individual was 3 (IQR: 0–10) out of a total of 52 weeks.

The distribution of the weekly maximum changes in individual RHR was calculated as follows. First, we calculated for each individual *i* and for each week *w* with at least four RHR measurements the range of RHR, namely Δ_i_^w^, defined as the difference between the maximum and the minimum RHR in that week. Then, for each individual, we calculated the maximum range over all the available weeks, Δ_i_^(max)^ = max_w_ Δ_i_^w^. Similarly, the weekly expected changes were calculated by computing Δ_i_^w^ for all weeks with at least four measurements. Then, for each individual we calculated the expected range over all the available weeks, Δ_i_^(exp)^ = median_w_ Δ_i_^w^.

To better understand the frequency of transient increases in RHR among individuals, we evaluated the number of episodes of unusual increases in RHR that lasted for at least three consecutive RHR measurements in 5 days. We defined an episode of unusual increase when the RHR increased by more than 2 standard deviations above the average of the individual’s previous 20 RHR measurements.

The 1-year trends for three subjects have been smoothed with a 7-day triangular smoothing function, to improve readability. The mean RHR has been modified and set to the value of 67 bpm to protect the identities of the individuals.

## Results

### Population characteristics

The 92,457 included individuals wore a device for at least 20 hours/day for a median of 320 days (IQR, 242–438) over a maximum possible monitoring period of 730 days, providing a total of 32,741,021 daily RHR values. Average age was 45.8 ± 14.4 years; 63% were women; average BMI was 28.4 ± 5.8 kg/m^2^; and average sleep duration was 6.8 ± 0.8 hours/day.

### Resting heart rate

**Fig 2:**
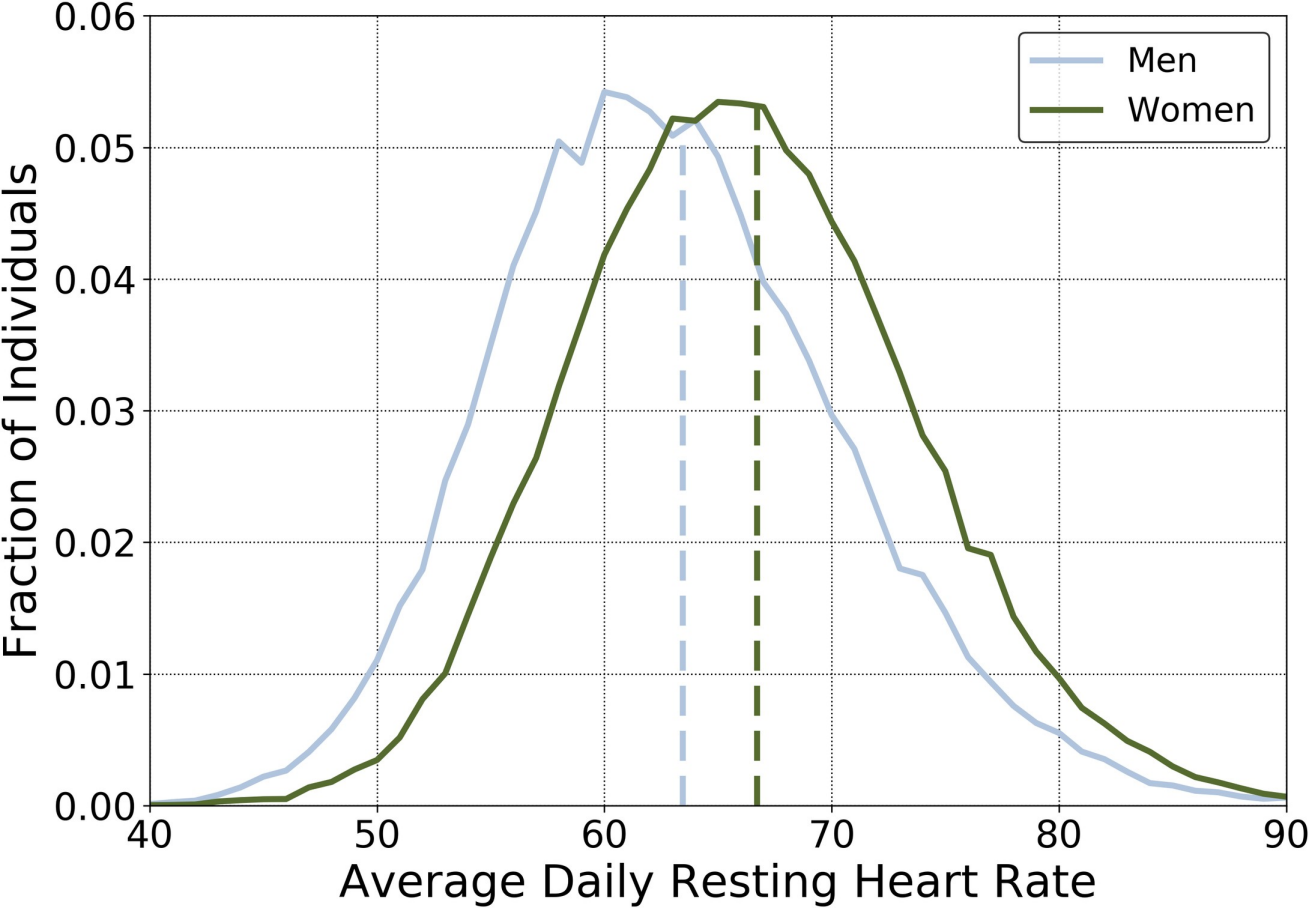
Distribution of average daily resting heart rates. The average daily RHR for 57,836 individual women (green line) and 34,621 men (blue line). The overall mean for each group is indicated by the dashed lines.

**Fig 3:**
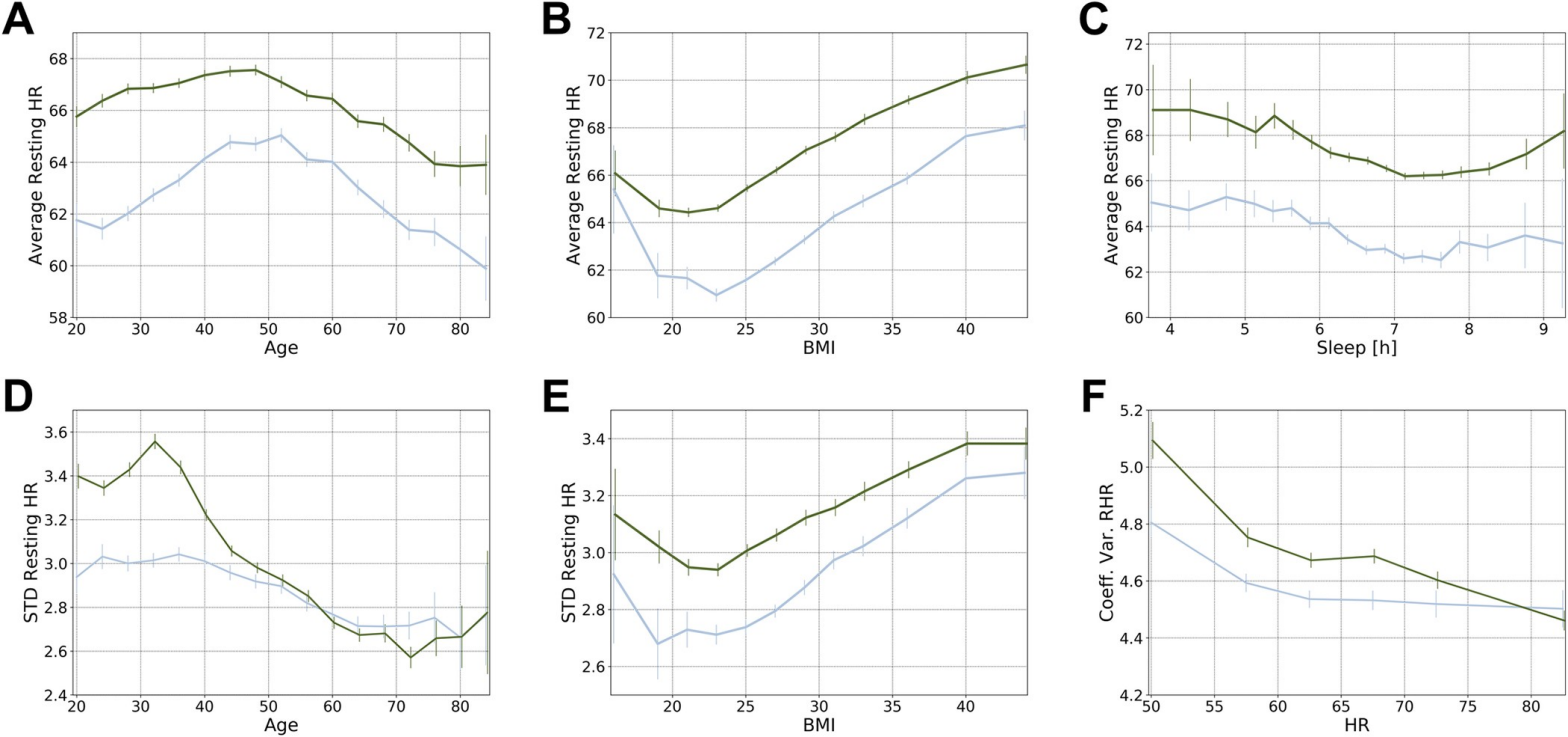
**Influence of age (A), body-mass index (BMI) (B), and hours of sleep (C) on average daily RHR, influence of age (D) and body-mass index (BMI) (E) on the standard deviation (STD), and influence of the mean RHR on the coefficient of variation (Coeff. Var.) of the RHR (F)**. The average, the standard deviation, and the coefficient of variation of the average daily RHR are shown for individual women (green) and men (blue line) in the cohort. Vertical lines indicate 95% confidence intervals in the estimation of the average for each subgroup.

The mean RHR for all individuals over all available monitored days was 65.5 ± 7.7 bpm, with a minimum and maximum average RHR for each individual ranging between 39.7 and 108.6 bpm, respectively. We observed that 95% of men had an RHR between 50 and 80 bpm, while the corresponding range for women was between 53 and 82 bpm (Fig 2). Women had a significantly higher RHR in all age brackets (Fig 3A). Across age groups, average RHR increased until approximately 50 years of age and then began a downward trend. Resting HR and BMI appeared to have a U-shaped relationship, with the lowest RHR associated with a BMI of 21 for women and 23 for men (Fig 3B). Average daily sleep duration was also associated with RHR, with the minimum RHR registered for both men and women who slept an average of 7–7.5 hours per night (Fig 3C). Differences in RHR as a function of age, BMI, and sleep duration were all statistically significant (p<0.01).

Interindividual variability in RHR was explained only in part by sex, age, BMI, and sleep duration. Sex alone was responsible for 4% of the variance in the average RHR among individuals, while sex and age together accounted for 6% of the variance; sex and BMI, for 7%; and sex and sleep duration, for 5%. Sex, age, BMI, and sleep duration together accounted for no more than 10% of total interindividual variability.

### Variability in individual resting heart rate

The average relative variability, expressed as standard deviation, in individuals’ daily RHR over time was 3.03 (3.10 for women, 2.90 for men). Age was associated with significant differences (Fig 3D), with subjects older than 60 years showing the lowest variability. Women of childbearing age showed greater variability in RHR compared with their male counterparts, with a peak in the early 30s. This difference disappeared by age 50.

Fluctuations in RHR were lowest for people with a BMI between 20 and 25 kg/m^2^, while both underweight and overweight participants demonstrated greater variability in HR (Fig 3E). Finally, to investigate if variability is influenced by the average RHR, we calculated the coefficient of variation, defined as the ratio between the standard deviation and the average RHR of an individual. The coefficient of variation increased as the average RHR dropped below 60 bpm, while it was almost constant (at least for men) in those with an average RHR >60 bpm (Fig 3F).

**Fig 4:**
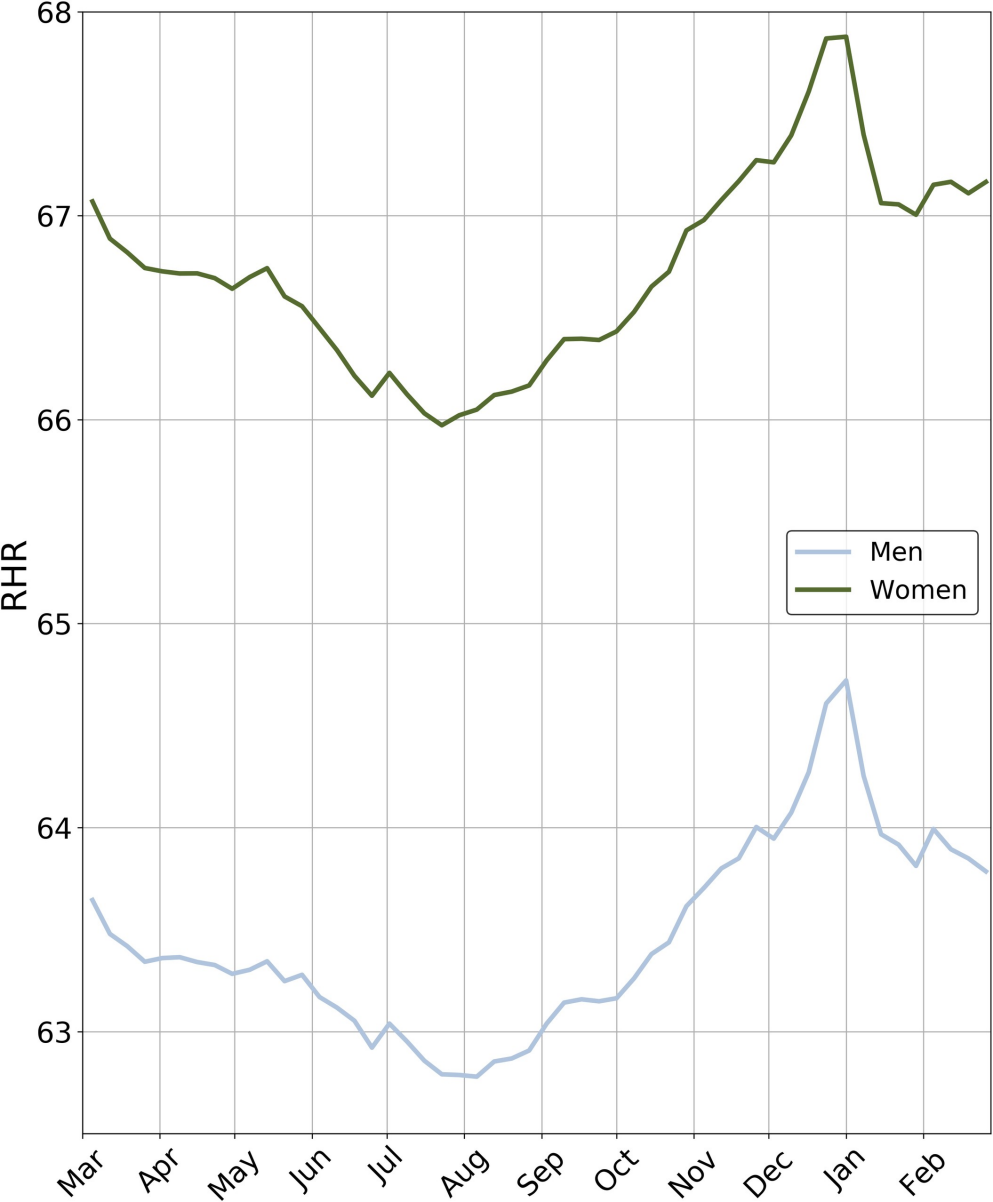
Weekly variation in overall average RHR for women (green line) and men (blue line) over 1 year. For each individual, and for each of the 52 weeks in a year, the average RHR was calculated considering all available data and interpolating over weeks with no data.

Over the course of a year, a small but significant seasonal change was observed. The change in the population’s average RHR was 2 bpm over the year. The RHR peaked in the first week of January for both men and women, after which the average RHR decreased to the yearly minimum at the end of July (Fig 4). After this minimum, the average RHR steadily increased until the end of the year.

**Fig 5:**
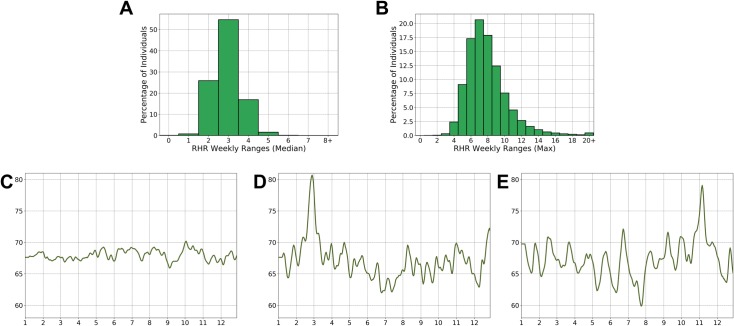
**Distribution of the median (A) and maximum (Max) (B) difference in the resting heart rate (RHR) within 1 week and examples of longitudinal RHR variations in three women (C, D, E) over 1 year.** For each individual, the median (A) and the maximum difference (B) in RHR over a week has been calculated considering all weeks with at least four RHR measurements. In the longitudinal examples, (C) shows a stable RHR for a woman over age 60 years; (D) a single episode of increased RHR in a woman between 50 and 60 years old; and (E) recurring monthly variability in a woman of childbearing age.

The RHR also fluctuated for individuals over the short term, i.e., a single week. Most subjects had a median weekly fluctuation in RHR of only 3 bpm (Fig 5A), and the maximum weekly fluctuation for ~80% of individuals was <10 bpm (Fig 5B). The number of episodes of RHR unusual increases (greater than two standard deviations above their average RHR, lasting at least three consecutive RHR measurements in 5 days) in individuals ranged from 0 to 17 over 2 years, with a median of three episodes over this period (IQR, 2–5).

To demonstrate some patterns of short-term variability over time, examples are shown of a stable RHR for a woman over 60 years old (Fig 5C), a single episode of increased RHR in a woman between 50 and 60 years old (Fig 5D), and recurring monthly variability in a woman of childbearing age (Fig 5E).

## Discussion

An estimated 20% of consumers in the U.S. now possess a smart watch or fitness band capable of passively and unobtrusively measuring continuous HR over long periods [27]. This new, unique source of individual physiologic data provides an opportunity to explore and define what could be considered a new vital sign of the digital age—individual trajectories in RHR. Indeed, a longitudinal view of the changes in RHR might prove to be a rich source of individual health information, not only for cardiovascular health but also for pulmonary status, infectious disease detection, reproductive health, and possibly more. The present study describes the largest cohort of longitudinal RHR data to date, with more than 92,000 subjects and RHR measured for over 32,000,000 days.

There are two key findings from this study. First, average RHR over time varies widely—from 40 to 109 bpm—between individuals. This variation is minimally associated with individual characteristics such as age and sex. In contrast to this large interindividual variation in average RHR, the second key finding was that intraindividual changes over time were an order of magnitude narrower. Individual variability was associated with several factors. First, we observed the presence of a seasonal trend, with the RHR of individuals changing by an average of 2 bpm, from a minimum in the midsummer to a maximum at the beginning of the year. Second, intraindividual variability in RHR is associated with sex (women had greater variability than did men) and age (women of childbearing age had significantly greater variability than similarly aged men or older women). This finding is supported by other studies—current fertility guidance applications can identify menstrual cycle phases based on daily changes tracked in RHR [28]. Intraindividual variability in RHR is also associated with BMI (variability was increased in subjects with a BMI >30 kg/m^2^). This is interesting, as another large study has noted that increases in RHR over a 2- to 10-year period are associated with an increased incidence of diabetes [29].

Beyond RHR variability over the long term, we also detected changes over the short term, i.e., within a week, that were most commonly only ~3 bpm. However, subjects also had infrequent episodes (median of three, over up to 2 years) when their RHR increased by more than two standard deviations above their average RHR. While we have no clinical data and thus cannot associate these episodes with any changes in health, others have found that increases in RHR preceded a formal diagnosis of acute infection and asthma exacerbations [30, 31]. From these early studies, it is worth considering that a rising RHR may serve as an early warning sign of a physiologic change. The ability to detect early acute illnesses, such as infections, and early exacerbations of chronic diseases remains a promising avenue to explore.

### Strengths and limitations

This retrospective study analysed the largest dataset of RHR to date. Beside the large number of participants, it has a unique length (2 years) and depth (measurements obtained daily in normal living conditions) that allowed the analysis of day-to-day variations not previously observed. Smaller studies in selected populations have shown that day-to-day changes in individual RHR are associated with infections, asthma exacerbations, and menstrual cycle phases. These early results suggest that day-to-day changes in RHR could be the first true, individualized “digital vital sign” as it is only now possible to measure thanks to wearable sensor technologies. A longitudinal view of the changes in RHR may prove to be a rich source of individual health information, and our manuscript is the first to describe the extent of inter- and intra-individual changes in RHR over a prolonged period of time.

Our work is best understood in the context of its limitations. Our study used HR data from a commercial-grade wearable device, which may not consistently capture heart rate as accurately as an electrocardiogram, even if in other studies it has been shown that this difference is quite small. However, this limitation seems to pertain primarily during activity and times of rapid heartbeat [20, 24, 32] and therefore would be unlikely to impact our findings, as the RHR was primarily calculated during periods of sleep.

RHR was determined based on a proprietary algorithm developed by the manufacturer, which varies based on whether the device is being worn during sleep. We attempted to minimize any artificial variability in RHR determination by considering RHR values as valid only if the device had been worn for at least 20 hours on that day.

The population included in this study was composed of persons who owned a wearable device measuring RHR. This cohort is not likely to be representative of the total socioeconomic and demographic diversity of the U.S., despite its unprecedented size.

In conclusion, in this large cohort that wore a fitness device over a median 320 days, individuals had a daily RHR that was normal for them but could differ from another individual’s normal by nearly 70 bpm. Within individuals, however, RHR was much more consistent over time, with a small but significant seasonal trend, and importantly, with detectable discrete and infrequent episodes outside their norms. Starting from the results of this explorative paper, a prospective study will be needed to determine if the information in the daily RHR can be of value in identifying important physiologic changes in individuals, and if an early detection of these changes may be clinically actionable if promptly communicated to the individual.
